# Comparison of Sampling Methods for mtDNA Analysis for Identification of Predator Species Causing Wounds in Veterinary Forensic Cases

**DOI:** 10.3390/ani15243560

**Published:** 2025-12-11

**Authors:** Reina Ueda, Yuko Kihara, Aki Tanaka

**Affiliations:** Laboratory of Wildlife Medicine, Department of Veterinary Medicine, Nippon Veterinary and Life Science University, 1-7-1 Kyonan-cho, Musashino-shi 180-8602, Tokyo, Japan

**Keywords:** forensic veterinary medicine, dog bite injury, Mitochondrial s(mt)DNA, species identification, muscle tissue sampling, swab sample

## Abstract

In forensic veterinary investigations of suspicious animal deaths involving loss of body parts, it is essential to determine whether the wounds were caused by human intervention or by other animals. Detailed postmortem examination of the wounds, combined with DNA analysis to identify biological traces left by the offending animal, play a critical role in such cases. Two primary methods are available for sample collection: muscle tissue sampling from the wound site and surface swabbing of the wound area; however, comparative studies evaluating these two approaches remain limited. In this study, mitochondrial DNA (mtDNA) analysis was performed using both muscle tissue sampling and surface swabbing in three forensic veterinary necropsy cases involving suspicious animal deaths with wounds or missing body parts, with the aim of identifying the animal species responsible for the injuries. As a result, in Case 1, canine DNA was detected from the wound of a cat carcass using both sampling methods. In Case 2, canine DNA was detected only with the swab method from the wound of another cat carcass. In Case 3, feline DNA was detected only with the muscle tissue sampling method from the wound of a duck carcass. Muscle tissue sampling, which targets deep tissues, offers advantages in preserving DNA integrity and quality. In contrast, the surface swab sampling is non-invasive and enables sample collection over a broader surface area, making it a valuable complementary tool. Although this study is based on a small number of cases, the findings suggest that combining both sampling techniques in wound-associated DNA analysis can leverage the strengths of each method, thereby improving DNA detection efficiency and enhance the reliability of forensic examinations.

## Simple Summary

In this study, mitochondrial DNA (mtDNA) analysis was conducted to identify the animal species responsible for wounds observed in three cases (two cats and a duck) that underwent forensic veterinary necropsy. DNA samples were collected using two methods: muscle tissue sampling and surface swab sampling, with detection results compared between the two approaches. As a result, in Case 1, involving a cat carcass, canine DNA was detected using both sampling methods. In Case 2, also involving a cat carcass, canine DNA was detected only with the swab method. In Case 3, involving a duck carcass, feline DNA was detected only with the muscle tissue sampling method. Although this study is based on a limited number of cases, the combined application of both sampling techniques in wound-associated DNA analysis is expected to enhance detection efficiency and improve the reliability of forensic investigation by leveraging the complementary strengths of each method.

## 1. Introduction

Forensic veterinary medicine has recently garnered increased attention in the investigation of crimes and accidents involving animals, particularly in addressing issues such as animal abuse and illegal wildlife trades [1,2,3]. In practical forensic veterinary investigations, examinations including necropsy, toxicology, imaging, and histopathology are routinely conducted. Among these, DNA analysis plays a crucial role by enabling the identification of both victim and perpetrator animal species, as well as individual animals, thereby providing critical scientific evidence [4].

When missing body parts or wounds are present on a carcass, determining the mechanism of injury and identifying the responsible individual or animal becomes particularly important [5,6,7]. Possible causes of such injuries include accidents, attacks by other animals, human-inflicted wounds, or post-mortem damage. In these cases, DNA analysis to determine the animal species involved in wound formation serves as an important scientific basis. Mitochondrial DNA (mtDNA) markers are commonly used in forensic science because they can detect genetic variations that are effective for species identification and are more likely to be recovered from degraded or low-quantity samples due to their higher copy number compared to nuclear DNA [4,8,9].

Numerous reports, including those in the field of forensic science, have analyzed mtDNA derived from saliva remaining on wounds to identify the animal species involved in wound formation. To the best of our knowledge, no previous studies have reported the direct use of wound tissue as an extraction sample for salivary mtDNA, surface swab sampling remains the most widely reported sampling method for sample collection [10,11,12,13,14,15]. However, a major limitation of the surface swabbing approach is that environmental and physical factors may reduce the quantity of DNA present on the wound surface, potentially compromising detection sensitivity. Furthermore, very few studies have directly compared the DNA detection efficiency between these two sampling methods.

In this report, mtDNA was conducted to identify the animal species responsible for wound formation in three cases of forensic veterinary necropsies involving animal carcasses with missing body parts and wounds. Two sampling methods, muscle tissue sampling and surface swab sampling, were employed, and results were compared.

## 2. Cases

**Figure 1:**
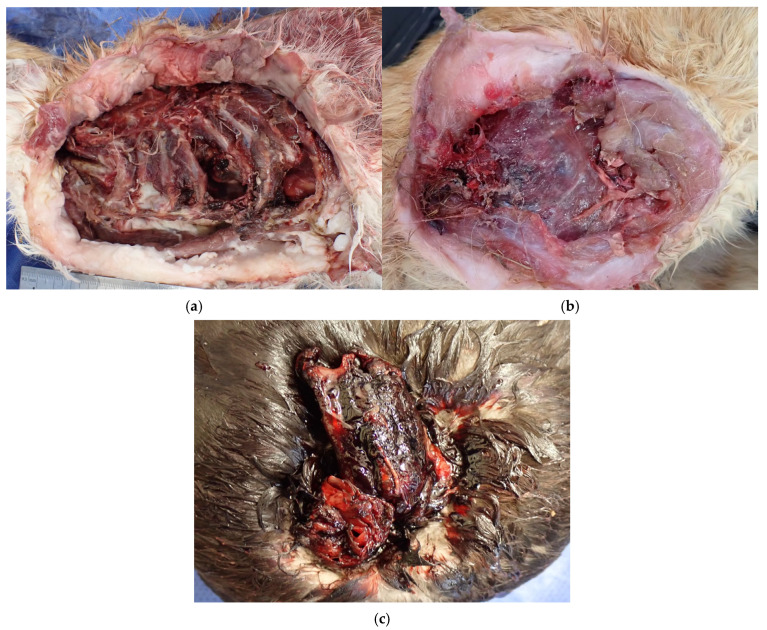
Wound sites in the three cases subjected to mtDNA analysis. (**a**) Case 1: Left thoracic region of a cat. The left forelimb is missing. The first to eighth ribs are exposed at the wound site, with hemorrhagic soft tissue remaining around the area. Multiple ribs exhibit fractures or loss of bone. (**b**) Case 2: Right thoracic region of a cat. The right forelimb is missing. Muscle tissue is exposed to the wound site, with surface muscles showing signs of crushing. Some areas surrounding soft tissue exhibit hemorrhage. Multiple rib fractures and bone loss were confirmed internally. (**c**) Case 3: Cervical region of a duck. The head and the eighth cervical vertebrae are missing. At the wound site, approximately 3 cm of cervical vertebrae and a small amount of surrounding soft tissue protrude. Extensive hemorrhage and blood coagulation are observed in the soft tissue.

All the cases involved suspicious animal deaths characterized by missing body parts and injuries. These cases were referred to forensic veterinary services by law enforcement authorities for necropsy and mtDNA analysis, with the aim of determining the mechanism of injury and assessing whether the wounds were of human origin. The subjects included in this study were selected based on necropsy findings that indicated or raised the suspicion of animal-inflicted trauma. The characteristics of each case are summarized in Table 1, and the anatomical sites from which mtDNA samples were collected are shown in Figure 1. The data were based on records from necropsies conducted at Nippon Veterinary and Life Science University, Tokyo, Japan.

## 3. mtDNA Analysis

### 3.1. Sampling

During gross examination at necropsy, care was taken to avoid contamination while collecting samples for DNA analysis. For the muscle tissue sampling method, sites within the wound suspected to bite marks were selected. The sample was obtained by scraping the muscle surface and underlying layers using a sterile scalpel, and a total of 25 mg was collected. For the surface swab method, sampling was performed after completion of muscle tissue collection. Depending on the dryness of the wound surface, either a sterile dry cotton swab or a swab moistened with TAE buffer was used. The swab was rubbed across the entire wound surface for approximately 10 s to collect the sample. All collected samples were stored at −20 °C until DNA extraction.

### 3.2. Selection of Target Animal Species for mtDNA Analysis

In Cases 1 and 2, the victim animals were cats, and the involvement of neighborhood-owned dogs was strongly suspected. Therefore, dogs were selected as the target species for mtDNA analysis. In Case 3, the victim animal was a duck. Given the absence of reports of wild animal sightings near the site and the occurrence of the event in a residential area within an urban setting, the species selected for mtDNA testing including masked palm civets a common urban wildlife species in Japan as well as cats [16] and raccoons [17], both of which have been reported to exhibit predatory behavior toward birds.

### 3.3. DNA Extraction

Genomic DNA was extracted from muscle tissue samples using the DNeasy Blood and Tissue Kit (QIAGEN, Hilden, Germany) in accordance with the manufacturer’s standard protocol, with a final elution volume of 100 μL Swab samples were processed using the QIAamp DNA Investigator Kit (QIAGEN, Hilden, Germany) following the manufacturer’s instructions, and DNA was eluted in a final volume of 50 μL. The concentration and purity of the extracted DNA were assessed using a NanoDrop Lite spectrophotometer (Thermo Fisher Scientific Inc., Waltham, MA, USA). All DNA extracts were stored at 2–5 °C until subsequent analyses.

### 3.4. Polymerase Chain Reaction (PCR) Amplification and Electrophoresis

For species identification of dogs, cats, and raccoons, species-specific primers previously designed based on reported mtDNA sequences were used (*Canis lupus familiaris* [18], *Felis catus* [18], and *Procyon lotor* [19]). For masked palm civets (*Paguma larvata*), a primer pair specifically designed at our laboratory was used. The primer pair for *P. larvata* was designed to amplify a fragment within the complete coding sequence (CDS) of the mitochondrial cytochrome b (cytb) gene (GenBank accession No. AB511054.1), corresponding to nucleotide positions 312–347 bp and 5–39 bp, respectively (Table 2). PCR amplification was performed using these primers, and the presence of target DNA amplification was confirmed by 2% agarose gel electrophoresis.

PCR amplification was conducted using a T100 Thermal Cycler (Bio-Rad, Hercules, CA, USA) according to the manufacturer’s protocol for KOD Fx Neo DNA polymerase (TOYOBO Co., Ltd., Osaka, Japan), with minor modifications. Each PCR reaction was prepared in a final volume of 25 μL containing 1× PCR Buffer for KOD Fx Neo, 0.4 mM dNTPs, 0.3 μM of each primer, <100 ng of template DNA, 0.5 U of KOD Fx Neo polymerase, and distilled water. The thermal cycling conditions consisted of an initial denaturation at 94 °C for 2 min, followed by 30 cycles of denaturation at 98 °C for 10 s, annealing at 61 °C for dog and raccoon, 70 °C for masked palm civet, and 63 °C for cat for 30 s, and extension at 68 °C for 1 min. PCR products were separated by electrophoresis on 2% agarose gels in 1× TAE buffer (Promega, Madison, WI, USA) containing loading buffer (Nippon Gene, Tokyo, Japan) and run at 100 V/cm for 20–25 min using a Mupid-2plus electrophoresis system (TaKaRa Co., Ltd., Shiga, Japan). After electrophoresis, gels were stained with ethidium bromide (EtBr; Nippon Gene, Tokyo, Japan) for more than 15 min, rinsed in distilled water for 5 min, and visualized under ultraviolet illumination to confirm the presence of amplification bands.

## 4. Results of mtDNA Analysis

**Figure 2:**
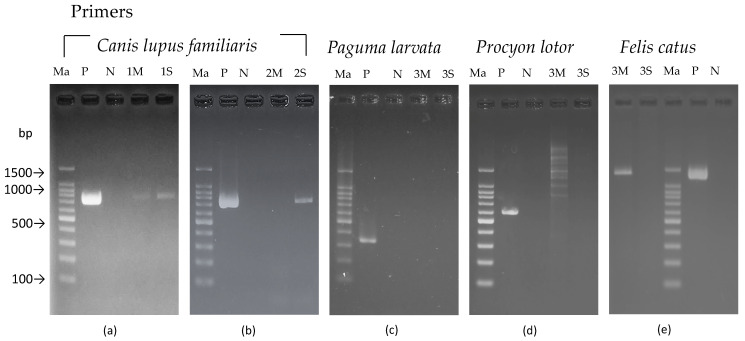
Electrophoresis results of the three cases analyzed in this study. (**a**) In Case 1, bands were detected from both sample collection methods for the dog test. (**b**) In Case 2, bands were detected only from the swab sample for the dog test. (**c**) In Case 3, no bands were detected in the masked palm civet test. (**d**) In Case 3, no bands were detected in the raccoon test. (**e**) In Case 3, bands were detected only from the muscle tissue sample in the cat test. Ma: DNA marker; P: positive control; N: negative control; 1–3M: muscle tissue samples from Cases 1–3; 1–3S: surface swab samples from Cases 1–3.

The electrophoresis images are shown in Figure 2, and the results of the DNA analysis are summarized in Table 3.

## 5. Discussion

Three forensic necropsy cases involving suspicious animal deaths with wounds and missing body parts or traumatic wounds were examined. To identify the animal species responsible for wound formation, mtDNA analysis was performed. In Case 1 (cat carcass), dog DNA was detected by both methods. In Case 2 (cat carcass), dog DNA was detected only in the surface swab sampling. In Case 3 (duck carcass), cat DNA was detected only from the muscle tissue sampling. Differences in mtDNA analysis results between the two sampling methods across the three cases were presumed to be influenced by multiple factors, including the amount of residual salivary DNA on the wound, the specific sampling area, and various external environmental conditions affecting the wound such as weather, insect activity, and ultraviolet (UV) light.

In Case 1, where dog mtDNA was detected in both muscle and swab samples from the cat carcass, it was suggested that salivary DNA was widely distributed on the soft tissue surface and even within the muscular layer. This finding may reflect the strong biting force characteristic of dog attacks, which allows the canine teeth to penetrate deeply into the victim’s tissue [20,21]. Furthermore, the mild postmortem changes observed in this case suggest that DNA degradation due to environmental factors such as temperature, humidity, and UV light exposure was limited. In contrast, in Case 2, a positive result was obtained only from the swab method. The relatively small amount of tissue collected in comparison with the overall wound area may have contributed to the increased likelihood of DNA non-detection in Case 2. Unlike the swab method, which allows DNA to be collected from the entire wound surface, the muscle tissue sampling method relies solely on the DNA adhering to a single tissue fragment per sample. Consequently, the amount of DNA obtained is inherently limited and subject to stochastic effects. Particularly in cases such as Case 3, where the wound area was small, the probability of detection is expected to be relatively higher, which may explain the differences in results observed among the cases. In Case 3, cat DNA was detected only in muscle tissue sampling. Since the surface swab sampling involves wiping the wound surface, it is more susceptible to environmental or physical factors that can remove or degrade surface DNA, which likely contributed to the negative result for the surface swab sampling.

The surface swab sampling offers the advantages of being non-invasive, rapid, and suitable for evidence collection at the crime scenes or from carcasses and contribute to the preservation of evidence. Moreover, since the sampling area can be set relatively large, the likelihood of DNA detection increases. However, this method is more susceptible to contamination and environmental influences around the wound, which may compromise result reliability. In contrast, the muscle tissue sampling method, though invasive and limited in the amount of material obtainable per sample, allows for the collection of soft tissue from deeper within the wound. Compared with surface swab samples, such tissues are more likely to contain higher-quality DNA, representing a significant advantage. From these perspectives, combining both the swab and muscle tissue sampling methods in wound DNA analysis may complement each other’s strengths, thereby improving the overall detection rate and reliability of the results. During muscle tissue collection, it is crucial to photograph and document the wound thoroughly before sampling to preserve detailed records of its original condition. Furthermore, as observed in Case 3, where tests for both raccoon and masked palm civet yielded negative results, it is important to consider the possibility of bites by non-target species or DNA degradation due to external environmental factors. Therefore, negative DNA results do not necessarily exclude the involvement of a particular animal species. It is also important to recognize that trace DNA recovery from wounds can vary depending on the investigator’s sampling expertise and the post-sampling storage conditions [22]. Thus, the standardization of sampling procedures remains a key challenge. In this study, amplification was confirmed by simple gel electrophoresis; however, sequencing analysis is recommended for definitive species identification.

Although this study was based on three cases, which is limitation of this study, future research will aim to accumulate additional data under more diverse environmental conditions and across various animal species. Such efforts are expected to further validate the applicability of wound DNA analysis, compare detection efficiencies between sampling methods, and contribute to forensic veterinary investigations. Ultimately, this may improve the accuracy of animal attack assessments and promote the establishment of reliable DNA analysis protocols based on appropriate sample collection.

## 6. Conclusions

In this study, three forensic necropsy cases involving suspicious animal deaths with wounds and missing body parts or traumatic wounds were examined to identify the animal species responsible for the injuries. mtDNA analysis was performed using two sampling methods: the muscle tissue sampling and the wound surface swab sampling. Although this report is based on the analysis of three cases, the combined use of both methods in wound DNA analysis is expected to complement their respective advantages, thereby improving detection efficiency and the overall reliability of forensic examinations.

## Figures and Tables

**Table 1:** Characteristics of the cases.

	Case 1	Case 2	Case 3
Animal species	Cat	Cat	Duck
Scene of Discovery	Yard inside the dormitory	On Residential Premises	Within the driveway of a private residence
Main missing part	From the left forelimb to the left thoracic region of skin	Right forelimb	Head
Bloodstains at the scene	No	No	Yes
Identification of the missing part near the carcass discovery site	No	Bone fragments	No
Characteristics around missing part	Left thoracic region missing (16 cm × 12 cm), rib exposure, fracture, and hemorrhage	Right thoracic region missing (5 cm × 10 cm), rib fracture, hemorrhage	Neck region missing (4 cm × 4 cm), missing from the Head to the 8th cervical vertebra, hemorrhage
Skin and soft tissue morphology	Irregular	Irregular	Irregular
Other major injuries	Perforation of the thigh, injuries to thoracic and abdominal cavities	Abdominal perforation, injuries to thoracic and abdominal cavities	Fractures of both wings and femurs, injuries to thoracic and abdominal Cavities
Presumed cause of death	Exsanguination	Exsanguination	Exsanguination
Presumed mechanism of injury	Bite trauma, scavenging	Bite trauma, scavenging	High-energy trauma, scavenging of the neck
Animal species presumed to be involved by necropsy findings	Dog	Dog	Unknown

**Table 2:** The primer sets employed for species identification in this study.

Scientific Name	Forward	Sequence (5′–3′)	Reverse	Sequence (5′–3′)	Size Range (Base Pair, bp)
*Canis lupus familiaris*	S19_Dog_F	GCCCAACTAACCCCAAACTTA [18]	S20_Dog_R	GGTTAACAATGGGGTGGATAAG	755
*Procyon lotor*	PLO-L15997	CCATCAGCACCCAAAGCT [19]	PLO-CRL1	CGCTTAAACTTATGTCCTGTAACC	608
*Paguma larvata*	hakubisinF	CCAACATTCGAAAATCTCACCCACTCGCTAAAATT (designed at out laboratory)	hakubisinR	CCAATGTTTCATGTCTCTGAAAAGGTATATGAACC	343
*Felis catus*	S39_Cat_F2	TTATCACACCCACAAGAGGA [18]	S40_Cat_R2	GTAGTACTTTCGACTGGTTAG	1391

**Table 3:** Results of DNA analysis.

Case	Wound Site	Sample	Electrophoresis Results	Positive Species
1	Left thoracic region	Muscle	Positive	Dog
		Swab	Negative	Dog
2	Right thoracic region	Muscle	Negative	
		Swab	Positive	Dog
3	Neck	Muscle	Positive	Cat
		Swab	Negative	

## Data Availability

This article contains all data found in this study. Further details or information can be directed at the corresponding author.

## Abbreviations

The following abbreviations are used in this manuscript:

mtDNA	Mitochondria DNA
PCR	Polymerase Chain Reaction
UV	Ultraviolet
